# Contrasting signals of cardiovascular health among natriuretic peptides in subjects without heart disease

**DOI:** 10.1038/s41598-019-48553-y

**Published:** 2019-08-20

**Authors:** Timothy C. R. Prickett, Janet K. Spittlehouse, Allison L. Miller, Yusmiati Liau, Martin A. Kennedy, Vicky A. Cameron, John F. Pearson, Joseph M. Boden, Richard W. Troughton, Eric A. Espiner

**Affiliations:** 1 Departments of Medicine, University of Otago, Christchurch, New Zealand; 2 Psychological Medicine, University of Otago, Christchurch, New Zealand; 3 Pathology and Biomedical Science, University of Otago, Christchurch, New Zealand; 4 Biostatistics and Computational Biology Unit, University of Otago, Christchurch, New Zealand

**Keywords:** Predictive markers, Cardiovascular biology, Risk factors

## Abstract

Natriuretic Peptides (NP) are important in maintaining normal cardiac and metabolic status and have been used to predict cardiovascular events. Whether plasma concentrations of NP products within the normal range reflect cardio-metabolic health is unknown. Plasma NTproANP, NTproBNP and NTproCNP and their bioactive counterparts were measured in a random sample of 348 community dwellers aged 49–51 yr without heart disease and associations sought with established vascular risk factors, echocardiographic indices and a genetic variant previously linked with BNP. Stratified by sex, each of ten vascular risk factors were positively associated with NTproCNP whereas associations with NTproBNP and NTproANP were all negative. In both sexes, higher plasma NTproCNP was associated with higher arterial elastance, lower LV stroke volume and lower LV end diastolic volume. Exactly opposite associations were found with plasma NTproBNP or NTproANP. Sex specific differences were identified: positive association of NTproBNP with LV end systolic volume and the negative association with LV elastance were found only in males. The genetic variant rs198358 was independently associated with NTproBNP but not with NTproANP. In conclusion, higher NTproCNP is likely to be an adaptive response to impaired LV relaxation whereas genetic factors likely contribute to higher NTproBNP and improved cardio-metabolic health at midlife.

## Introduction

Since their discovery, increasing evidence shows that Atrial Natriuretic Peptide (ANP) and B-type Natriuretic Peptide (BNP), acting via the specific receptor NPR1, play a critical role in maintaining cardiovascular health, in addition to regulating lipogenesis and energy balance^1^. Further, in detecting impaired cardiac function^2^, plasma concentrations of these peptides are increasingly used in community studies as predictors of incident cardiovascular events. However the degree to which plasma concentrations relate to indices of cardio-metabolic health in community subjects without heart disease requires further study – preferably in settings where comprehensive analysis of established vascular risks and cardiac function are made at the time of sampling. In this context, associative studies have the potential to reveal unexpected roles of NPs otherwise difficult to determine in humans.

Compared to ANP and BNP, much less is known concerning C-type Natriuretic Peptide (CNP). CNP, acting via its receptor NPR2, is a paracrine/autocrine growth factor which drives postnatal endochondral bone growth^3,4^, has recognised anti-inflammatory and anti-proliferative actions in the vascular intima^5^, and is thought to have important roles in regulating pressure and flow in the microcirculation^6^. CNP is also expressed in myocardial tissues though its contribution to cardiac performance in humans remains unclear^7^. Plasma concentrations of bio-active CNP are low and close to limits of detection in keeping with rapid clearance and degradation at source but a processed bio-inactive component of tissue proCNP (aminoterminal proCNP, NTproCNP) can be readily measured in plasma^8^. In contrast to the cardiac secreted peptides, concentrations of CNP products in plasma are unlikely to be subject to regulation. However, consistent with upregulation of CNP gene expression in the vascular endothelium^9^ in response to vascular stress^10^, we find positive associations of plasma NTproCNP with a range of vascular risk factors (VRF)^8,11^, and with higher blood pressure in pregnancy^12^. Collectively these findings suggest that vascular – and possibly cardiac stress – contribute to circulating levels of NTproCNP in adults. Although favourable effects of ANP/BNP signalling on lipogenesis and metabolic risk have been documented in community studies^13,14^, whether higher levels of these peptides within the normal reference range also associate with favourable effects on cardiac performance is unknown. Clarifying the relationships of natriuretic peptides (NP) with cardiovascular health – for example at mid-life when risk of events is increasing – is therefore a key issue particularly in the light of the reported positive association of BNP peptides with increased cardio-vascular risk in older subjects^15^.

Here this issue is addressed in a study of 348 community dwellers aged 49–51 yr – after excluding those with known heart disease – recruited as part of the Canterbury Health Ageing and Life Course (CHALICE) health survey conducted in Christchurch New Zealand during 2010–2013^16^. Because of the known interactions among bioactive forms^17^, and the singularly low levels of CNP in plasma, we used the aminoterminal forms of each NP in these comparative associative studies. For completeness, the bioactive forms were also measured.

## Results

### Demographic clinical and laboratory findings

Of the 404 participants (50% female), 78% reported NZ European ethnicity and 15.2% reported Māori or Pacific ethnicity. Thirty subjects had previous heart disorders (myocardial infarction (14 subjects), angina (9 subjects), cardiac arrhythmia (23 subjects),heart failure (5 subjects)) and therefore were excluded from analyses. In a further 26 subjects, blood samples were unavailable for analysis. Details of the remainder (348 subjects) are shown in Table 1. At study recruitment, 10.5% were receiving hypotensive therapy, and 9% were taking lipid lowering drugs. 14% were active smokers. In 45 subjects echocardiography was not performed. With respect to sex differences, significantly higher waist, stroke volume, LV mass, LVEDV, LVESV, hs-Troponin, plasma albumin, urate, creatinine, eGFR, GGT, lipids, hematocrit (HCT) and NTproCNP values were found in males whereas LV and arterial elastance and plasma NTproBNP and NTproANP were all significantly lower than those of females. Concentrations of bioactive forms showed the same sex specific changes as their N terminal forms (Table 1).

**Table 1:** Demographic, clinical and laboratory findings. Values are medians (interquartile range).

	All Subjects (n = 348)	Females (n = 192)	Males (n = 156)	*P*
Age (yr)	50.8 (50.4–51.3)	50.9 (50.4–51.5)	50.6 (50.2–51.3)	**0.002**
Height (cm)	169 (164–177)	165 (161–168)	178 (173–183)	<**0.001**
Weight (kg)	81 (70–93)	75 (64–89)	86 (78–94)	<**0.001**
BMI	27 (24–31)	27 (24–33)	27 (25–30)	0.56
Waist (cm)	94 (84–102)	88 (80–101)	96 (91–103)	<**0.001**
Systolic BP (mm Hg)	131 (120–141)	129 (118–139)	133 (124–142)	**0.023**
Diastolic BP (mm Hg)	81 (74–88)	80 (73–87)	82 (76–89)	**0.036**
Heart rate (bpm)	65 (58–73)	68 (61–76)	61 (54–68)	<**0.001**
Pulse pressure (mm Hg)	49 (43–57)	49 (42–57)	49 (44–56)	0.58
Stroke volume* (ml/m^2^)	41 (35–47)	38 (34–44)	46 (40–49)	<**0.001**
LA area* (cm^2^/m^2^)	8.2 (7.3–9.1)	8.2 (7.1–9.1)	8.4 (7.4–9.1)	0.39
LV mass* (g/m^2^)	99 (83–115)	91 (78–103)	107 (94–124)	<**0.001**
LVEDV* (ml/m^2^)	64 (55–72)	58 (51–66)	70 (63–76)	<**0.001**
LVESV* (ml/m^2^)	22 (19–26)	21 (17–23)	24 (21–28)	<**0.001**
LVEDWS (kdynes/cm^2^)	34 (29–40)	33 (28–40)	35 (31–40)	0.052
Ejection Fraction (%)	65 (62–68)	65 (63–69)	64 (61–67)	0.07
LV elastance* (mmHg/ml)	6.0 (5.1–7.3)	6.5 (5.4–7.8)	5.6 (4.9–6.7)	<**0.001**
Arterial elastance* (mmHg/ml)	3.3 (2.8–3.8)	3.5 (2.9–4.0)	3.0 (2.7–3.5)	<**0.001**
E/A	1.1 (1.0–1.4)	1.1 (1.0–1.4)	1.1 (1.0–1.4)	0.96
E/e’	8.2 (6.9–10)	8.3 (7.1–10.4)	7.9 (6.5–9.5)	**0.010**
hs-Troponin (ng/L)	4.8 (3.3–6.6)	3.8 (3.0–5.0)	6.1 (4.8–8.0)	<**0.001**
PRA (nmol/L/hr)	0.9 (0.6–1.3)	0.8 (0.6–1.2)	0.9 (0.7–1.4)	**0.019**
Aldosterone (pmol/L)	139 (100–182)	140 (100–185)	136 (100–174)	0.51
Plasma albumin (g/L)	42 (40–43)	41 (39–43)	42 (41–44)	<**0.001**
Plasma urate (mmol/L)	0.31 (0.26–0.37)	0.27 (0.23–0.32)	0.36 (0.31–0.40)	<**0.001**
Plasma creatinine (umol/L)	80 (74–88)	75 (70–79)	88 (83–94)	<**0.001**
eGFR (ml/min/1.73 m2)	79 (73–86)	75 (71–81)	84 (78–90)	<**0.001**
Urine total protein creatinine ratio (g/L)	3.0 (1.0–6.6)	3.0 (1.0–7.4)	3.0 (1.0–6.0)	0.98
Gamma glutamyltransferase (U/L)	23 (16–36)	18 (13–28)	28 (21–41)	<**0.001**
Fasting Glucose (mmol/L)	5.0 (4.7–5.4)	4.9 (4.6–5.3)	5.1 (4.8–5.5)	**0.003**
Fasting Insulin (pmol/L)	45 (31–75)	45 (30–74)	47 (32–78)	0.78
HOMA	1.4 (0.9–2.4)	1.4 (0.9–2.3)	1.5 (1.0–2.5)	0.38
HbA1c (mmol/mol)	38 (35–40)	38 (34–40)	38 (36–40)	0.47
Total Cholesterol (mmol/L)	5.4 (4.8–5.9)	5.3 (4.8–5.9)	5.5 (4.8–6.0)	0.22
Total Cholesterol/HDL ratio	4.0 (3.3–4.7)	3.7 (3.1–4.4)	4.4 (3.8–5.0)	<**0.001**
Triglycerides (mmol/L)	1.2 (0.8–1.6)	1.1 (0.8–1.5)	1.3 (1.0–1.8)	<**0.001**
Haemoglobin (g/L)	138 (130–149)	131 (125–137)	150 (141–154)	<**0.001**
Haematocrit	0.41 (0.39–0.44)	0.40 (0.38–0.41)	0.44 (0.42–0.45)	<**0.001**
ANP (pmol/L)	9.2 (7.1–12.4)	10.1 (7.7–13.5)	8.1 (6.4–10.8)	<**0.001**
NTproANP (nmol/L)	0.35 (0.27–0.46)	0.38 (0.31–0.50)	0.30 (0.25–0.40)	<**0.001**
BNP (pmol/L)	3.8 (3.1–5.2)	4.3 (3.3–5.9)	3.6 (2.8–4.4)	<**0.001**
NTproBNP (pmol/L)	11.2 (6.7–20)	14.8 (9.6–25.1)	8.3 (5.4–14.3)	<**0.001**
CNP (pmol/L)	0.28 (0.20–0.40)	0.25 (0.18–0.32)	0.34 (0.24–0.46)	<**0.001**
NTproCNP (pmol/L)	16 (14–19)	14 (13–16)	17 (15–21)	<**0.001**

*Values indexed to body surface area.

Abbreviations: LA, left atrium; LV, left ventricle; LVEDV, left ventricular end-diastolic volume; LVESV, left ventricular end-systolic volume; LVEDWS, LV end diastolic wall stress; E/A ratio of early to late ventricular filling velocities; E/e’, ratio of transmitral Doppler early filling velocity to tissue Doppler early diastolic mitral annular velocity; Hs Troponin, high sensitivity Troponin; HOMA, homeostatic model assessment of insulin resistance; eGFR, estimated glomerular filtration rate; HbA1c, hemoglobin A1c.

### Univariate associations of natriuretic peptides with risk factors and echocardiography findings

#### Aminoterminal peptides

**Figure 1:**
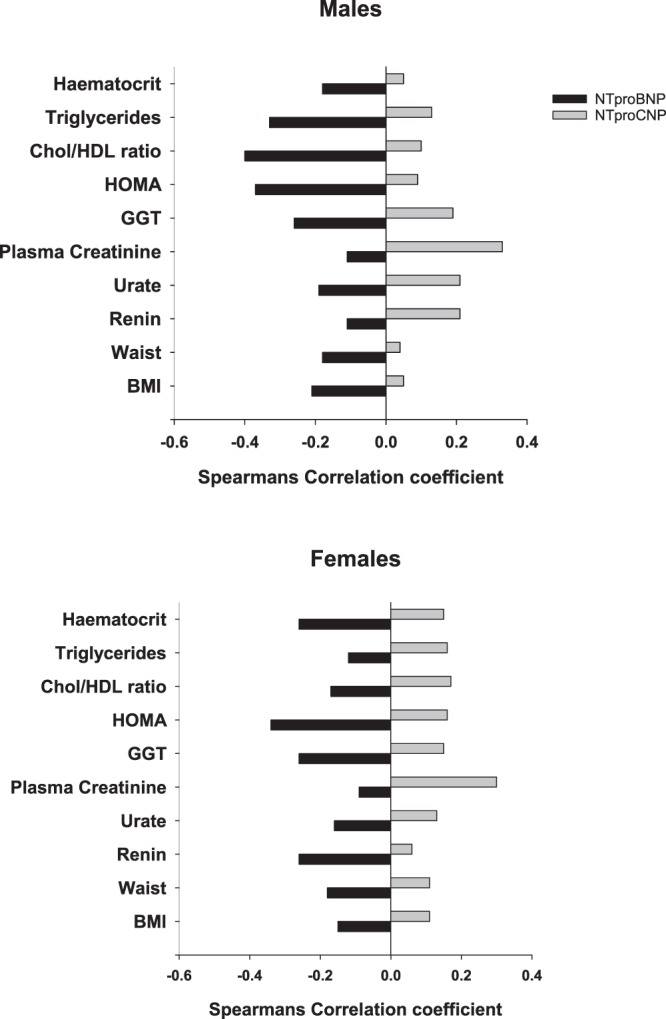
Comparison of correlation coefficients of NTproBNP and NTproCNP with cardiovascular risk factors. Coefficients of ≥0.15 (P < 0.05) or ≥0.26 (P < 0.001) were significant.

As shown in Table 2, across all subjects, significant inverse associations of NTproBNP with a wide range of variables were identified whereas associations of these with NTproCNP were mostly significantly positive. When analysed separately by sex, significant negative associations of NTproBNP with established vascular risk factors (BMI, waist, urate, GGT, HOMA, Total Cholesterol, Chol/HDL ratio and HCT) were found in both males and females. Negative associations of renin and aldosterone were confined to females, and association of TG with NTproBNP was only observed in males. Associations of NTproANP were broadly similar to those found with NTproBNP in both sexes except for associations with renin (NTproANP stronger in males) and urate (NTproBNP stronger in males). The negative association of both peptides with TG was stronger in males. With respect to plasma NTproCNP, significant positive associations with creatinine and GGT were found in both sexes; hs TnT, HOMA, Chol/HDL, TG and HCT were significant only in females, and renin and urate only in males. As shown in Fig. 1, associations of ten established VRFs with NTproBNP and NTproCNP were all in opposite directions in both males and females.

**Table 2:** Associations of vascular and metabolic indices with amino terminal natriuretic peptides

	All Subjects (n = 348)			Females (n = 192)			Males (n = 156)		
	NTproANP	NTproBNP	NTproCNP	NTproANP	NTproBNP	NTproCNP	NTproANP	NTproBNP	NTproCNP
BMI	**−0.20** ^†^	−0.16*	0.06	**−0.24** ^†^	−0.15*	0.11	−0.17*	**−0.21** ^†^	0.05
Waist	**−0.28** ^†^	**−0.27** ^†^	0.17*	**−0.26** ^†^	−0.18*	0.11	−0.15	−0.18*	0.04
Systolic BP	−0.05	−0.10	0.08	−0.05	−0.11	0.12	0.02	0.02	−0.06
Diastolic BP	−0.13	−0.12*	0.09	−0.13	−0.10	0.05	−0.08	−0.06	0.05
Pulse pressure	0.02	−0.03	0.00	0.01	−0.07	0.07	0.07	0.06	−0.15
hsTroponin	−0.11*	−0.15*	**0.28** ^†^	0.05	0.04	0.15*	−0.03	−0.01	0.04
Renin	**−0.26** ^†^	**−0.22** ^†^	0.15*	**−0.25** ^†^	**−0.26** ^†^	0.06	**−0.29** ^†^	−0.11	**0.21** ^†^
Aldosterone	−0.12*	−0.08	0.03	**−0.24** ^†^	**−0.21** ^†^	0.09	−0.04	0.04	−0.02
Plasma albumin	**−0.21** ^†^	**−0.26** ^†^	0.17*	−0.15*	**−0.23** ^†^	0.04	−0.18*	−0.13	0.16
Plasma urate	**−0.26** ^†^	**−0.34** ^†^	**0.33** ^†^	**−0.19** ^†^	−0.16*	0.13	−0.07	-0.19*	**0.21** ^†^
Plasma creatinine	**−0.21** ^†^	**−0.32** ^†^	**0.48** ^†^	−0.02	−0.09	**0.30** ^†^	− 0.02	−0.11	**0.33** ^†^
eGFR	−0.12*	−0.09	−0.08	0.02	0.09	**−0.30** ^†^	0.02	0.11	**−0.33** ^†^
ɣ glutamyltransferase	**−0.33** ^†^	**−0.36** ^†^	**0.29** ^†^	**−0.27** ^†^	−**0.26**^†^	0.15*	**-0.26** ^†^	**−0.27** ^†^	0.19*
HOMA	**−0.40** ^†^	**−0.33** ^†^	0.15*	**−0.43** ^†^	**−0.34** ^†^	0.16*	**−0.37** ^†^	**−0.37** ^†^	0.09
HbA1c	**−0.13**	−0.14	0.14	**−0.25** ^†^	−0.19	0.18	0.05	−0.05	0.06
Total Cholesterol	**−0.18** ^†^	**−0.23** ^†^	0.12*	−0.09	−0.22*	0.11	**−0.24** ^†^	−0.20*	0.10
Chol/HDL ratio	**−0.33** ^†^	**−0.35** ^†^	**0.25** ^†^	**−0.21** ^†^	−0.17*	0.17*	**−0.37** ^†^	**−0.40** ^†^	0.13
Triglycerides	**−0.32** ^†^	**−0.28** ^†^	**0.24** ^†^	−0.18*	−0.12	0.16*	**−0.39** ^†^	**−0.33** ^†^	0.13
Haemoglobin	**−0.36** ^†^	**−0.40** ^†^	**0.33** ^†^	**−0.25** ^†^	**−0.29** ^†^	0.11	**−0.24** ^†^	−0.18*	0.05
Haematocrit	**−0.35** ^†^	**−0.37** ^†^	**0.31** ^†^	**−0.23** ^†^	**−0.26** ^†^	0.15*	**−0.28** ^†^	−0.18*	0.04

*P < 0.05.

^†^*P* < *0.001*.

**Figure 2:**
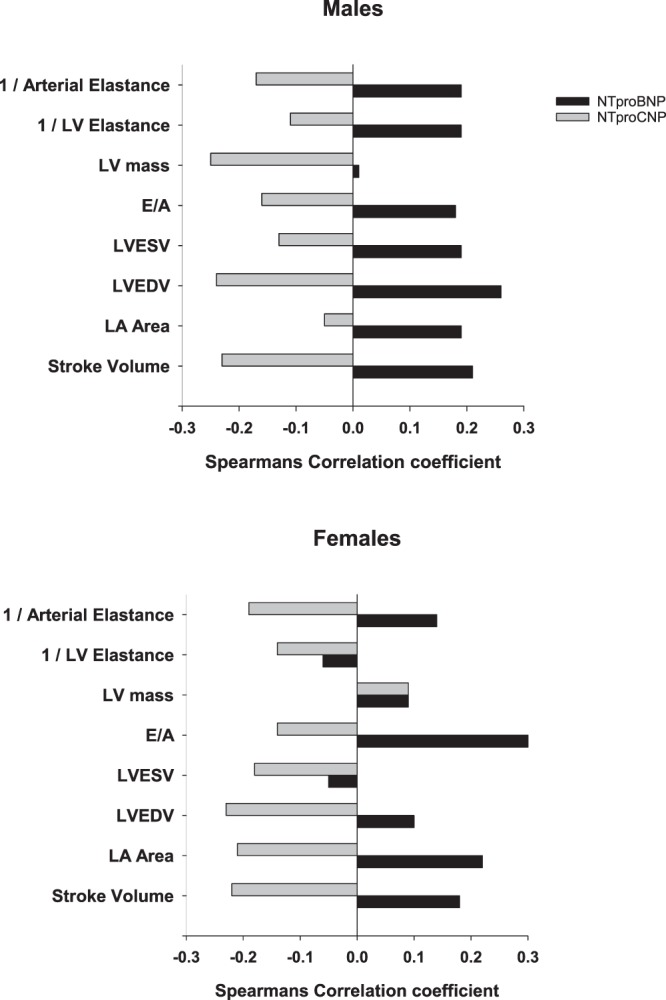
Comparison of correlation coefficients of NTproBNP and NTproCNP with transthoracic echocardiographic indices. The inverse of arterial elastance and LV elastance is presented to unify the directionality of risk. Coefficients ≥0.18 (P < 0.05) or ≥0.30 (P < 0.001) were significant.

Associations of echocardiography values with respective peptides are shown in Table 3 and Fig. 2. In both males and females, stroke volume was positively correlated with plasma NTproBNP and negatively with NTproCNP. No significant associations of any NP were observed with LV end diastolic wall stress. In both males and females positive associations of plasma NTproBNP with LA area and E/A ratio were found whereas positive associations with LVEDV and LVESV were identified only in males. Similarly the significant negative associations of NTproBNP with LV and arterial elastance were confined to males. With respect to NTproANP, associations were similar to those found for NTproBNP in both sexes excepting those with LVEDV and LV mass in females (NTproANP stronger in both) and with LVESV (positive associations were identified with ANP, NTproANP, BNP and NTproBNP, but only in males). Positive associations of both NTproANP and NTproBNP with ejection fraction were stronger in females. Consistent with the negative associations of stroke volume with NTproCNP, positive associations of NTproCNP with arterial elastance were found in both sexes. Negative associations of NTproCNP with LVEDV in both sexes, with LA area and LVESV in females, and with LV mass in males were also noted. Again, for r values exhibiting significance, most of these were opposite to those found with NTproBNP (Fig. 2).

**Table 3:** Associations between echocardiography parameters and amino terminal natriuretic peptides including a subset of subjects with normal indices of diastolic function.

	All Subjects			Females			Males		
	NTproANP	NTproBNP	NTproCNP	NTproANP	NTproBNP	NTproCNP	NTproANP	NTproBNP	NTproCNP
**Entire cohort**									
Stroke volume^‡^ (303)	0.11	0.02	−0.02	**0.26** ^†^	0.18*	**−0.22** ^†^	**0.28** ^†^	0.21*	**−0.23** ^†^
LA area^‡^ (265)	**0.27** ^†^	**0.17** ^†^	0.11	**0.31** ^†^	0.22*	−0.21*	**0.31** ^†^	0.19*	−0.05
LVEDV^‡^ (303)	0.06	−0.02	−0.01	0.18*	0.10	**−0.23** ^†^	**0.27** ^†^	**0.26** ^†^	**−0.24** ^†^
LVESV^‡^ (303)	−0.03	−0.08	0.02	0.02	−0.05	−0.18*	0.15	0.19*	−0.13
LV mass^‡^ (276)	−0.00	−0.12*	0.13*	**0.22** ^†^	0.09	0.09	0.07	−0.01	**−0.25** ^†^
Ejection Fraction (294)	**0.22** ^†^	**0.19** ^†^	−0.03	**0.23** ^†^	**0.21** ^†^	0.01	0.17*	0.08	−0.02
E/A (309)	**0.30** ^†^	**0.25** ^†^	−0.14*	**0.36** ^†^	**0.33** ^†^	−0.14	**0.24** ^†^	0.18*	−0.16
E/e’ (301)	0.03	0.03	0.00	0.03	0.05	0.05	−0.08	−0.11	0.07
LVWEDWS^‡^ (291)	0.03	0.06	−0.12*	−0.01	−0.06	−0.11	−0.02	0.14	−0.05
LV elastance^‡^ (303)	0.02	0.05	0.01	0.01	0.06	0.14	−0.14	−0.19*	0.11
Arterial elastance^‡^ (303)	−0.12*	−0.04	0.06	−0.19*	−0.14	0.19*	**−0.23** ^†^	−0.19*	0.17*
**Normal diastolic function**									
Stroke volume^‡^ (246)	0.10	0.01	0.06	**0.26** ^†^	0.16	−0.16	**0.27** ^†^	0.20*	−0.15
LA area^‡^ (219)	**0.24** ^†^	0.16*	−0.06	**0.25** ^†^	0.23*	−0.22*	**0.31** ^†^	0.15	0.07
LVEDV^‡^ (246)	0.02	−0.05	0.09	0.17	0.07	−0.15	**0.24** ^†^	0.22*	−0.16
LVESV^‡^ (246)	−0.07	−0.11	0.10	0.00	−0.06	−0.13	0.10	0.14	−0.07
LV mass^‡^ (221)	−0.03	−0.16*	0.14*	**0.26** ^†^	0.14	0.01	0.01	−0.09	**−0.25** ^†^
Ejection Fraction (238)	**0.24** ^†^	**0.22** ^†^	−0.03	0.22*	0.19*	0.06	0.20*	0.15	0.01
E/A (250)	0.20*	**0.21** ^†^	−0.09	**0.31** ^†^	**0.31** ^†^	−0.08	0.21*	0.16	−0.16
E/e’ (250)	−0.01	0.00	0.00	0.08	0.10	−0.02	−0.15	−0.13	0.04
LVEDWS^‡^ (237)	0.02	0.06	−0.11	0.01	−0.04	−0.08	−0.05	0.11	−0.06
LV elastance^‡^ (246)	0.07	0.08	−0.09	0.06	0.07	0.07	−0.10	−0.15	0.02
Arterial elastance^‡^ (246)	−0.11	−0.04	−0.03	−0.18*	−0.13	0.11	−0.24*	−0.21*	0.08

*P < 0.05.

^†^*P < 0.001*.

^‡^Indexed to body surface area. Bracketed values indicate number of subjects.

Since some subjects may have subclinical diastolic dysfunction, a sub analysis was undertaken after exclusion of those with abnormal E/A ratio (<0.85, n = 23) and or E/e’ (>12, n = 52)^18^. In the remainder with normal diastolic function (n = 234), relationships of NPs with E/A were unchanged. However in both sexes the negative association of NTproCNP with LV volumes, LV stroke volume and the positive association with arterial elastance were all attenuated (Table 3) whereas those of NTproANP and NTproBNP were largely unchanged. The negative association of NTproCNP with LA area in females (opposite to that of NTproANP or NTproBNP), and the negative association of NTproCNP with LV mass in males remained significant in these subjects with normal indices of diastolic function.

#### Bioactive peptides

Associations of ANP, BNP and CNP with vascular and metabolic indices are shown in Supplementary Table 1, and with echo parameters in Supplementary Table 2. Overall, while directionality was retained, similar to aminoterminal forms, associations of ANP or BNP with vascular or with echo data were weaker or not significant. With respect to CNP, association with vascular and metabolic indices were also weaker compared to those of NTproCNP. However in contrast to NTproCNP (significant only in men), CNP was positively linked to renin in both sexes. As expected, associations of CNP with creatinine were not significant in contrast to the highly significant positive associations of NTproCNP in both sexes. As noted for the ANP and BNP associations with echo findings, those of CNP were also reduced – stroke volume and arterial elastance excepted – compared to those of NTproCNP.

### Multivariable linear regression analyses

The relative strengths of risk factors associated with concentrations of each individual peptide were assessed separately using multivariable regression analysis. In addition to the 10 factors identified by univariate analysis (Fig. 1), the initial model also incorporated sex, echocardiography parameters (LA-area, E/A ratio and LV Stroke volume), and smoking. The results show that for NTproBNP, sex (P < 0.001), HOMA-IR (P = 0.002) and E/A ratio (P = 0.013) remained independently significant associations (Table 4 Model 2). For NTproANP, sex (P < 0.001), LA-area (P < 0.001), HOMA-IR (P = 0.002), Chol/HDL ratio (P = 0.021), E/A ratio (P = 0.023) and creatinine (P = 0.044) remained independently significant associations (Table 4 Model 1). For NTproCNP, creatinine (P < 0.001), LV Stroke volume (P = 0.002), GGT (P = 0.002), sex (P = 0.004) renin (P = 0.016) and smoking (P = 0.04) remained significant (Table 4, Model 4).

**Table 4:** Multivariable Linear Regression Analyses.

	B	SE(B)	β	t	Sig
**Model 1 Dependent variable NTproANP**					
(Constant)	−0.57	0.10		−5.7	**0.000**
Sex	−0.109	0.024	−0.33	−4.6	**0.000**
Creatinine	0.002	0.001	0.14	2.0	**0.044**
HOMA	−0.099	0.032	−0.20	−3.1	**0.002**
LA area*	0.026	0.007	0.21	3.8	**0.000**
E/A	0.197	0.086	0.13	2.3	**0.023**
Total Chol/HDL ratio	−0.022	0.010	−0.14	−2.3	**0.021**
Renin	−0.024	0.013	−0.10	−1.8	0.070
**Model 2 Dependent variable NTproBNP**					
(Constant)	1.30	0.14		9.6	**0.000**
Sex	−0.181	0.042	−0.28	−4.3	**0.000**
HOMA	−0.182	0.059	−0.19	−3.1	**0.002**
LA area*	0.025	0.013	0.11	1.9	0.060
E/A	0.42	0.17	0.14	2.5	**0.013**
Urate	−0.54	0.28	−0.13	−1.9	0.056
**Model 3 Dependent variable NTproBNP**					
(Constant)	1.27	0.13		9.6	**0.000**
Sex	−0.175	0.041	−0.27	−4.2	**0.000**
HOMA	−0.188	0.058	−0.20	−3.2	**0.001**
LA area*	0.025	0.013	0.10	1.9	0.061
E/A	0.44	0.16	0.15	2.7	**0.008**
Urate	−0.61	0.28	−0.15	−2.2	**0.029**
rs198358	0.106	0.034	0.16	3.1	**0.002**
**Model 4 Dependent variable NTproCNP**					
(Constant)	0.879	0.058		15.3	**0.000**
Sex	0.044	0.015	0.21	2.9	**0.004**
Creatinine	0.003	0.001	0.30	4.6	**0.000**
LV Stroke Volume*	−0.002	0.001	−0.17	−3.2	**0.002**
ɣ glutamyltransferase	0.063	0.020	0.16	3.1	**0.002**
Renin	0.010	0.004	0.12	2.4	**0.016**
Current Smoker	0.031	0.015	0.10	2.1	**0.041**

*Indexed to body surface area.

NTproANP, NTproBNP, NTproCNP, ɣ glutamyltransferase, HOMA and E/A data were log10-transformed to satisfy parametric assumptions. The coefficient of multiple determination (R^2^) for each of the four models was 0.32, 0.28, 0.31 and 0.31 respectively.

### Effect of absolute plasma NTproBNP concentration on vascular risk

Anticipating that the negative association of NTproBNP with risk will reduce or change direction at higher plasma concentrations, significance of associations with a composite index of risk (employing internationally recognized cutoff values for systolic blood pressure, lipids and HOMAR-IR)^11^ was calculated in each sex according to selected cut-off values. In males, using NTproBNP cut-off values from <13 through <25 pmol/L, negative associations remained significant with vascular risk (range of r values −0.44 to −0.49). However for NTproBNP concentrations >25 pmol/L, the association with vascular risk was positive. In females, where plasma NTproBNP levels are approximately 2-fold those of males (Table 1), similar negative association with risk prevailed at concentrations from <13 through <25 pmol/L. Values exceeding these showed no significant relationship to risk.

### Cardiovascular events

In the 5 year follow-up period after the initial study visit, when study bloods were collected and cardiac echocardiography was performed, 5% of the study participants were admitted to hospital for one or more cardiovascular events. Baseline NTproBNP or NTproCNP concentrations for male or females subsequently hospitalised were not significantly different from values of study participants remaining free from cardiovascular events.

### Genetic polymorphisms

Since a significant positive association of the SNP rs198358 with plasma NTproBNP was found in our previous study – where higher levels of the peptide were also associated with reduced risk – the same analysis was made in the CHALICE cohort. A significant positive association with levels of plasma NTproBNP (P = 0.008) was found using a dominant model. Including this variant in the multivariable regression model (Table 4, Model 3), backwards stepwise regression revealed that sex (P < 0.001), HOMA-IR (P = 0.001), rs198358 SNP (P = 0.002), E/A ratio (P = 0.008) and urate (P = 0.029) remained independent predictors of plasma NTproBNP. The frequency (0.23) of the minor allele rs198358 in the current study was similar to a previous study (0.24)^11^ and reported frequency in the European population 1000 Genomes (0.25). No influence of rs198358 on plasma NTproANP level was observed (P = 0.64).

## Discussion

Associative studies of NPs with cardio-metabolic health at midlife are important in view of the increasing evidence of their role in maintaining cardiovascular health throughout life. In this first study of all three NPs in subjects without history of heart disease, we provide new evidence of links of paracrine CNP with arterial stiffening and myocardial structural integrity. In both sexes, higher plasma NTproCNP was associated with higher arterial elastance and reduced LV stroke volume and lower LV end diastolic volume. Exactly the opposite associations were found with endocrine ANP/BNP: higher concentrations were associated with lower arterial elastance, enhanced LV stroke volume and higher LVEDV. Similar opposing relationships were found in 10 separate metabolic indices recognized as VRFs (Fig. 1). Overall trends for lower associations of the bioactive forms with both vascular and cardiac indices likely reflects their shorter half-life and greater lability. The SNP variant was found to be independently associated with plasma NTproBNP, suggesting that genetic factors are likely to increase measures of cardiac performance and reduce metabolic risk factors by increasing endocrine BNP. The opposing relationships of CNP are likely to reflect compensatory (protective) responses to vascular and myocardial stress. By eliminating the influence of age – which variously impacts not only vascular and cardiac health but also affects hormonal activity^19^ – these new findings illuminate relationships of NPs with function at a critical phase in the course of evolving cardiovascular degenerative disorders. Importantly, they also emphasise the need to distinguish beneficial (physiological) concentrations of BNP in community studies from those associated with impaired cardiac function.

Although *in vitro* and *ex vivo* studies point to important roles of CNP/NPR2 signaling in modulating myocardial inflammation and vascular tone^1,5^, evidence of its importance in humans has been difficult to obtain^7^. In previous studies, which have included subjects with overt heart disease, plasma bioactive CNP concentration was **positively** correlated with LV volumes^20^, or with a pathological cardiovascular phenotype, LV dysfunction and raised BNP^21^. However, we find at midlife that higher NTproCNP (and CNP) is linked to reduced stroke volume, reduced LV volumes and higher arterial elastance. These findings suggest that an early (adaptive) change in CNP gene expression occurs within cardiac tissues before impaired cardiac function is clinically evident – possibly evoked by increases in peripheral vascular resistance and change in myocardial structural integrity. Relevant here are findings from rodents^22^ and *ex vivo* studies^23^ showing the important lusitropic role of the CNP/NPR2 pathway in promoting LV relaxation. This is further supported by the sub-analysis where these links of CNP with LV volumes and arterial elastance were lost after excluding subjects with presumed diastolic dysfunction. Importantly, sex differences were also found. Negative relationships between NTproCNP and LA area and LVESV were stronger in females, and in males (but not females) higher NTproCNP was associated with lower LV mass.

Complementing these positive associations of CNP with cardiac stress were similar links with a range of metabolic markers affecting cardio-vascular health. Strong positive links of renal function and plasma GGT with NTproCNP were evident in both sexes whereas positive links with other metabolic factors (HOMA, Chol/HDL ratio, TG and HCT) were stronger in women. These associations of NTproCNP with GGT, lipids and insulin activity are likely to be adaptive (compensatory) responses to disordered lipogenesis^24^ including hepatic fat accumulation. The findings connect with evidence in rodents where upregulation of CNP expression in hepatic vascular endothelium occurs in response to high fat diets and with evidence from genetic models where enhanced CNP expression in the hepatic endothelium inhibits inflammation and restores insulin activity in hepatic steatosis^25^. The strong positive association of creatinine with plasma NTproCNP (but not with CNP) in both sexes contrasts with the lack of associations with either NTproANP or NTproBNP which are similarly cleared by the kidney^26^ and suggests that the kidney itself increases the contribution to plasma levels of NTproCNP as renal function is threatened. Evidence that the CNP gene is expressed in renal tissues, is upregulated in response to renal inflammation^27^ and that renin is an independent predictor of NTproCNP (current study) collectively support an important reno-protective role even in settings of normal glomerular function – findings not previously described in humans.

Previous reports of relationships between ANP or BNP with measures of cardiac performance have mostly focused on subjects with cardiac impairment. Less is known of these associations in subjects free of overt cardiac disease^7^. In this context it needs to be emphasized that in health there is a progressive age dependent decrease in LV stroke volume and LV volumes, and increase in LV mass^28^ along with increase in LV and arterial elastance in both sexes. Aligning with these age related changes are increases in plasma ANP and BNP. In the current study of subjects – all the same age and with most echo values and plasma NPs within the normal reference range – unexpected sex specific relationships are revealed which are likely to be relevant to the differing evolution of cardiac pathology in the two sexes^29^. Specifically in both sexes, at midlife higher concentrations of NTproBNP and NTproANP are associated with improved cardio-vascular performance – higher stroke volume, LA area and LV volumes, higher rates of early atrial filling and lower arterial elastance. Notably, stratifying by sex reveals that in males the positive association of NTproBNP and NTproANP with LVESV (also found for the bioactive counterparts), and the negative association with LV elastance (similarly in bioactive forms), are not evident in females whereas links of both peptides with EF were stronger in females. Together with the positive link of NTproANP with LV mass (only found in females), these observations are consistent with previous studies showing increased cardiac remodeling in females^29^ in whom both LV and arterial elastance are increased compared to values in males (Table 1).

Numerous studies from the community show that ANP and/or BNP products in plasma are negatively associated with all components of the metabolic syndrome, other than elevated blood pressure^13^. Previous studies have also linked a number of genetic variants affecting ANP^14^ and/or BNP^30^ products in plasma with components of the metabolic syndrome, whereas other variants are associated with lower blood pressure^31^ as well as improved cardiovascular health outcomes and lower cardiovascular mortality^32^. In the latter sequential study of 11,361 subjects aged 45–65 years, a closely related variant (rs198389) was associated with lower blood pressure and higher levels of plasma NTproBNP at each age examined, although at older age the beneficial effect on blood pressure was reduced. The variant we identified (rs198358) is also an independent positive predictor of NTproBNP in both young and older age groups but associated with lower diastolic blood pressure only in young adults. However, the same variant does not predict plasma concentrations of NTproANP as shown in the current study.

The underlying molecular events connecting lower NTproBNP with the diverse array of vascular risk factors measured in this study is likely to be mediated by reduced ANP/BNP receptor (NPR1) signaling – activation of which inhibits adipogenesis and increases mitochondrial efficiency^24^. In light of this evidence, an alternative hypothesis – that acquired disorders of lipogenesis reduce cardiac secretion of BNP peptides^33^ – seems less plausible.

Our study has some limitations. Associations of natriuretic peptides with cardiovascular and metabolic health were made at a single time point. Presumably, this and the small number of subjects studied, and the short period of follow up, explain the lack of benefit on health outcomes. Recent work suggests that androgens negatively affect BNP products in both sexes^34^. Although we analysed associations in each sex separately, and have eliminated the influence of age (which reduces androgens in both males and females), effects of androgens on relationships of ANP/BNP with risk cannot be excluded but are likely to be small. The possibility that the positive links of NTproCNP with impaired cardiac and metabolic health reflect resistance to the peptide’s actions in tissues^4^ – rather than an adaptive response to vascular stress – cannot be excluded. Finally although participants were free of heart disease and were asymptomatic, increase in BNP in some may be due to subtle increases in LV hypertrophy and or end diastolic pressure. However their exclusion would be expected to strengthen the positive association of NTproBNP with cardiac performance. Notwithstanding these limitations, the remarkably consistent results in young subjects with the current, more detailed, assessment at mid-life add cogency to our findings.

## Subjects and Methods

Details of the Canterbury Health Ageing and Life Course (CHALICE) study have been published previously^16^. CHALICE is a multidisciplinary community study of 404 participants aged 49–51 yr recruited randomly from the electoral roll. Ethical approval for the CHALICE study was obtained from the Upper South A Regional Ethics Committee (URA/10/03/021) and conducted in accordance with the Declaration of Helsinki. All procedures were conducted at the University of Otago, Christchurch and Canterbury District Health Board facilities by trained health professionals during the years 2010–13. Details of echocardiography and genotyping are listed in the Supplementary Methods. After obtaining informed written consent, the following assessments relevant to this report were performed: health history (including demographics, ethnicity, chronic morbidities and medications, tobacco consumption (y/n, active at time of study); physical examination (including height, weight, BMI); heart function (including manual and automatic blood pressure, echocardiography); laboratory measures (including fasting urine and blood sampling and bio banking for DNA, routine biochemistry, and plasma storage for measurements of natriuretic peptides). Blood pressure of the non-dominant arm was measured manually in the sitting position after at least a 15 min period of rest using a mercury sphygmomanometer with a large cuff for arm circumference >33 cm.

Established humoral metabolic-cardiovascular risk factors were measured after an overnight fast and included the following:- total cholesterol, total cholesterol/HDL-cholesterol ratio (calculated), triglycerides (TG), HbA1c, glucose, insulin and HOMA-IR (homeostatic model assessment of insulin resistance)^35^. Also measured were less well recognized markers of vascular risk:- hs Troponin T (TnT)^36^, plasma renin activity (PRA) and aldosterone^37^, creatinine^38^, urate^39^, albumin^40^, gamma glutamyl transferase (GGT)^41^ and hematocrit^42^ using standard procedures. eGFR was calculated by the abbreviated MDRD equation^43^. Body surface area (BSA) was calculated using the Mosteller method (BSA = √(height × weight/3600). The protein/creatinine ratio was determined using an early morning urine. Plasma ANP, BNP, CNP, NTproANP, NTproBNP and NTproCNP were assayed as previously described^11,26^. Intra- and inter-assay coefficients of variations were as follows:- ANP (1.6% and 8.5% at 21 pmol/L); BNP (8.3% and 5.4% at 9.7 pmol/L); CNP (4.4% and 8.3% at 1.8 pmol/L); NTproANP (6.4% and 8.8% at 0.88 nmol/L); NTproBNP (4.9% and 5.9% at 83 pmol/L); NTproCNP (6.6% and 8.1% at 45 pmol/L).

Cardiovascular events (heart failure, angina, myocardial infarction, atrial fibrillation) in the period (up to 5 yr) following enrollment were recorded using information from the National Health Registry ICD codes, and hospital and general practitioner records when appropriate.

### Statistics

Differences between groups (males, females) were determined using Mann-Whitney U tests or Fisher’s exact test as appropriate. Because sex is known to affect cardiac and vascular phenotypes^29^ as well as plasma concentrations of NTproANP, NTproBNP and NTproCNP, univariate associations between natriuretic peptides and risk factors in each sex were separately assessed using Spearman’s correlation coefficients. Independence was assessed by multivariable regression analysis using backwards stepwise regression in a model comprising 10 separate variables found significant in univariate analyses. All model assumptions were assessed graphically and NTproANP, NTproBNP, NTproCNP, triglyceride, GGT, HOMA and E/A data were log10-transformed to satisfy parametric assumptions. All tests were two sided and statistical significance was assumed when P < 0.05.

## Supplementary information

Supplementary Information

## Data Availability

The datasets analysed during the current study are available from the corresponding author on reasonable request.

## References

[1] Kuhn M (2016). Molecular Physiology of Membrane Guanylyl Cyclase Receptors. Physiol Rev.

[2] Davis M (1994). Plasma brain natriuretic peptide in assessment of acute dyspnoea. Lancet.

[3] Nakao K (2015). The Local CNP/GC-B system in growth plate is responsible for physiological endochondral bone growth. Sci Rep.

[4] Espiner Eric, Prickett Tim, Olney Robert (2018). Plasma C-Type Natriuretic Peptide: Emerging Applications in Disorders of Skeletal Growth. Hormone Research in Paediatrics.

[5] Qian JY (2002). Local expression of C-type natriuretic peptide suppresses inflammation, eliminates shear stress-induced thrombosis, and prevents neointima formation through enhanced nitric oxide production in rabbit injured carotid arteries. Circ Res.

[6] Špiranec Katarina, Chen Wen, Werner Franziska, Nikolaev Viacheslav O., Naruke Takashi, Koch Franziska, Werner Andrea, Eder-Negrin Petra, Diéguez-Hurtado Rodrigo, Adams Ralf H., Baba Hideo A., Schmidt Hannes, Schuh Kai, Skryabin Boris V., Movahedi Kiavash, Schweda Frank, Kuhn Michaela (2018). Endothelial C-Type Natriuretic Peptide Acts on Pericytes to Regulate Microcirculatory Flow and Blood Pressure. Circulation.

[7] Matsuo A, Nagai-Okatani C, Nishigori M, Kangawa K, Minamino N (2018). Natriuretic peptides in human heart: Novel insight into their molecular forms, functions, and diagnostic use. Peptides.

[8] Prickett TC (2013). Impact of age, phenotype and cardio-renal function on plasma C-type and B-type natriuretic peptide forms in an adult population. Clin Endocrinol.

[9] Nakao K (2017). Endothelium-Derived C-Type Natriuretic Peptide Contributes to Blood Pressure Regulation by Maintaining Endothelial Integrity. Hypertension.

[10] Chun TH (1997). Shear stress augments expression of C-type natriuretic peptide and adrenomedullin. Hypertension.

[11] Prickett TCR (2018). New Insights into Cardiac and Vascular Natriuretic Peptides: Findings from Young Adults Born with Very Low Birth Weight. Clin Chem.

[12] Espiner EA, Prickett T, Taylor RS, Reid RA, McCowan LM (2015). Effects of pre-eclampsia and fetal growth restriction on C-type natriuretic peptide. Bjog.

[13] Olsen MH (2005). N-terminal pro brain natriuretic peptide is inversely related to metabolic cardiovascular risk factors and the metabolic syndrome. Hypertension.

[14] Cannone V (2013). The atrial natriuretic peptide genetic variant rs5068 is associated with a favorable cardiometabolic phenotype in a Mediterranean population. Diabetes Care.

[15] Wang TJ (2004). Plasma natriuretic peptide levels and the risk of cardiovascular events and death. N Engl J Med.

[16] Schluter PJ (2013). Canterbury Health, Ageing and Life Course (CHALICE) study: rationale, design and methodology. N Z Med J.

[17] Hunt PJ (1995). Interactions of atrial and brain natriuretic peptides at pathophysiological levels in normal men. Am J Physiol.

[18] Mitter SS, Shah SJ, Thomas JD (2017). A Test in Context: E/A and E/e’ to Assess Diastolic Dysfunction and LV Filling Pressure. J Am Coll Cardiol.

[19] Partridge L, Deelen J, Slagboom PE (2018). Facing up to the global challenges of ageing. Nature.

[20] Palmer SC, Prickett TC, Espiner EA, Yandle TG, Richards AM (2009). Regional Release and Clearance of C-Type Natriuretic Peptides in the Human Circulation and Relation to Cardiac Function. Hypertension.

[21] Sangaralingham SJ (2015). Circulating C-type natriuretic Peptide and its relationship to cardiovascular disease in the general population. Hypertension.

[22] Brusq JM, Mayoux E, Guigui L, Kirilovsky J (1999). Effects of C-type natriuretic peptide on rat cardiac contractility. Br J Pharmacol.

[23] Subramanian H (2018). Distinct submembrane localisation compartmentalises cardiac NPR1 and NPR2 signalling to cGMP. Nat Commun.

[24] Miyashita K (2009). Natriuretic peptides/cGMP/cGMP-dependent protein kinase cascades promote muscle mitochondrial biogenesis and prevent obesity. Diabetes.

[25] Bae CR, Hino J, Hosoda H, Miyazato M, Kangawa K (2018). C-type natriuretic peptide (CNP) in endothelial cells attenuates hepatic fibrosis and inflammation in non-alcoholic steatohepatitis. Life Sci.

[26] Prickett TC (2017). C-Type Natriuretic Peptides in Coronary Disease. Clin Chem.

[27] Surendran K, Simon TC (2003). CNP gene expression is activated by Wnt signaling and correlates with Wnt4 expression during renal injury. Am J Physiol Renal Physiol.

[28] Poppe K.K., Doughty R.N., Gardin J.M., Hobbs F.D.R., McMurray J.J.V., Nagueh S.F., Senior R., Thomas L., Whalley G.A., Aune E., Brown A., Badano L.P., Cameron V., Chadha D.S., Chahal N., Chien K.L., Daimon M., Dalen H., Detrano R., Akif Duzenli M., Ezekowitz J., de Simone G., Di Pasquale P., Fukuda S., Gill P.S., Grossman E., Hobbs F.D.R., Kim H.-K., Kuznetsova T., Leung N.K.W., Linhart A., McDonagh T.A., McGrady M., McMurray J.J.V., Mill J.G., Mogelvang R., Muiesan M.L., Ng A.C.T., Ojji D., Otterstad J.E., Petrovic D.J., Poppe K.K., Prendergast B., Rietzschel E., Schirmer H., Schvartzman P., Senior R., Simova I., Sliwa K., Stewart S., Squire I.B., Takeuchi M., Thomas L., Whalley G.A., Altman D.G., Perera R., Poppe K.K., Triggs C.M., Au Yeung H., Beans Picón G.A., Poppe K.K., Whalley G.A., Anderson T., Dyck J., Ezekowitz J.A., Chirinos J.A., De Buyzere M.L., Gillebert T.C., Rietzschel E., Segers P., Van daele C.M., Doughty R.N., Poppe K.K., Walsh H.A., Whalley G.A., Izzo R., De Luca N., Trimarco B., de Simone G., Chadha D.S., Goel K., Misra A., Chen P.-C., Chien K.-L., Lin H.-J., Su T.-C., Detrano R., Cameron V., Richards A.M., Troughton R., Mogelvang R., Skov Jensen J., Di Pasquale P., Paterna S., Akif Duzenli M., Hobbs F.D.R., Davies M.K., Davis R.C., Roalfe A., Calvert M., Davies M.K., Davis R.C., Freemantle N., Gill P.S., Lip G.Y.H., Kuznetsova T., Staessen J.A., Dargie H.J., Ford I., McDonagh T.A., McMurray J.J.V., Grossman E., Galasko G., Lahiri A., Senior R., Brown A., Carrington M., Krum H., McGrady M., Stewart S., Zeitz C., Blauwet L., Sliwa K., Stewart S., Dalen H., Moelmen Hansen H.E., Støylen A., Thorstensen A., Daimon M., Watanabe H., Yoshikawa J., Fukuda S., Kim H.-K., Leung N.K.W., Linhart A., Chahal N., Chambers J.C., Kooner J., Senior R., Davies J., Loke I., Ng L., Squire I.B., Aune E., Otterstad J.E., Leung D.Y., Ng A.C.T., Ojji D., Arnold L., Coffey S., d'Arcy J., Hammond C., Mabbett C., Lima C., Loudon M., Pinheiro N., Prendergast B., Reynolds R., Badano L.P., Muraru D., Peluso D., Dal Bianco L., Petrovic D.J., Petrovic J., Schvartzman P., Fuchs F.D., Katova T., Simova I., Kaku K., Takeuchi M., Boyd A., Chia E.M., Thomas L., Schirmer H., Angelo L.C., Pereira A.C., Krieger J.E., Mill J.G., Rodrigues S.L., Muiesan M.L., Paini A., Agabiti Rosei E., Salvetti M. (2015). Ethnic-Specific Normative Reference Values for Echocardiographic LA and LV Size, LV Mass, and Systolic Function. JACC: Cardiovascular Imaging.

[29] Hayward CS, Kalnins WV, Kelly RP (2001). Gender-related differences in left ventricular chamber function. Cardiovasc Res.

[30] Meirhaeghe A (2007). Association between the T-381C polymorphism of the brain natriuretic peptide gene and risk of type 2 diabetes in human populations. Hum Mol Genet.

[31] Newton-Cheh C (2009). Association of common variants in NPPA and NPPB with circulating natriuretic peptides and blood pressure. Nat Genet.

[32] Seidelmann, S. B. *et al*. An NPPB Promoter Polymorphism Associated With Elevated N-Terminal pro-B-Type Natriuretic Peptide and Lower Blood Pressure, Hypertension, and Mortality. *J Am Heart Assoc* **6**, 10.1161/JAHA.116.005257 (2017). 10.1161/JAHA.116.005257 PMC5533018 28341776

[33] Zhang H (2016). Regulation of B-type natriuretic peptide synthesis by insulin in obesity in male mice. Exp Physiol.

[34] de Lemos JA, Das SR (2019). Closing the Book on Androgens and Natriuretic Peptides. J Am Coll Cardiol.

[35] Gayoso-Diz P (2013). Insulin resistance (HOMA-IR) cut-off values and the metabolic syndrome in a general adult population: effect of gender and age: EPIRCE cross-sectional study. BMC Endocr Disord.

[36] Welsh P (2016). Prediction of Cardiovascular Disease Risk by Cardiac Biomarkers in 2 United Kingdom Cohort Studies: Does Utility Depend on Risk Thresholds For Treatment?. Hypertension.

[37] Farmer JA, Torre-Amione G (2001). The renin angiotensin system as a risk factor for coronary artery disease. Curr Atheroscler Rep.

[38] Manjunath G (2003). Level of kidney function as a risk factor for atherosclerotic cardiovascular outcomes in the community. J Am Coll Cardiol.

[39] Ndrepepa G (2018). Uric acid and cardiovascular disease. Clin Chim Acta.

[40] Chien SC, Chen CY, Lin CF, Yeh HI (2017). Critical appraisal of the role of serum albumin in cardiovascular disease. Biomark Res.

[41] Kunutsor SK, Apekey TA, Khan H (2014). Liver enzymes and risk of cardiovascular disease in the general population: a meta-analysis of prospective cohort studies. Atherosclerosis.

[42] Gijsberts CM (2015). Hematological Parameters Improve Prediction of Mortality and Secondary Adverse Events in Coronary Angiography Patients: A Longitudinal Cohort Study. Medicine (Baltimore).

[43] Levey AS (1999). A more accurate method to estimate glomerular filtration rate from serum creatinine: a new prediction equation. Modification of Diet in Renal Disease Study Group. Ann Intern Med.

