# Supplementary material for: Contrasting signals of cardiovascular health among natriuretic peptides in subjects without heart disease
Source: Sci Rep. 2019 Aug 20;9:12108. doi: 10.1038/s41598-019-48553-y (PMC6702214; doi:10.1038/s41598-019-48553-y)
Supplement: Supplementary file 1 — Supplementary Information [file 41598_2019_48553_MOESM1_ESM.docx]

**Supplementary Information**

**Manuscript Title:** Contrasting signals of cardiovascular health among natriuretic peptides in subjects without heart disease.

**Authors:** Timothy CR. Prickett, Janet K. Spittlehouse, Allison L. Miller, Yusmiati Liau, Martin A. Kennedy, Vicky A. Cameron, John F. Pearson, Joseph M. Boden, Richard W. Troughton and Eric A. Espiner.

**Supplementary Methods**

Echocardiography.

Standardized transthoracic echocardiography was performed using an iE33 ultrasound machine (Philips Life Healthcare). Standard M-mode measurements of LV dimensions and wall thickness and LA dimensions were made according to the recommendations of the American Society of Echocardiography. LV volume was measured by the Simpson modified biplane method and LA area and volume were estimated by the biplane area-length method. LVEDV and LVESV were indexed to body surface area to derive the indexed volumes (LVEDVi and LVESVi) and LV ejection fraction (LVEF). Mitral pulsed-wave Doppler velocities of early passive (E) and atrial (A) filling were obtained from the apical 4-chamber view with a 5-mm sample volume placed between the tips of the mitral leaflets. Tissue Doppler measurements of early diastolic myocardial relaxation (e′) was recorded from the lateral mitral annulus in the apical 4-chamber view with a 5-mm sample volume and with filters set to exclude high frequency signals and with gain minimized. Left ventricular (LV) elastance (systolic blood pressure/LV end systolic volume, LVESD) and arterial elastance (systolic blood pressure/ LV stroke volume) were calculated as detailed by Chantler et al (1). LV end diastolic pressure (LVEDP) was estimated using the following formula: LDEDP=0.54 mean arterial blood pressure x 0.54 x (1–ejection fraction) – 2.23 (2). LV end diastolic wall stress (LVEDWS) was calculated as follows: LVEDWS=0.334 x LVEDP x LV internal dimension at end-diastole / (wall thickness x (1 – wall thickness / LV internal dimension at end-diastole) (3).

Genotyping.

Genotyping of the SNP rs198358 was performed using a TaqMan® Assay (ID: C_2230494_10; Thermo Fisher Scientific, Waltham, MA USA). In a 96 well plate, 50 ng DNA of each sample was amplified with 0.25 unit Taq DNA polymerase (Fisher Biotec, Wembley, WA, Australia) in the presence of 1x PCR buffer, 0.2mM dNTP, and 5.5mM MgCl_2_ in 10 µL reaction using the Roche LightCycler® 480 real time polymerase chain reaction instrument. Genotypes were determined using the LightCycler® 480 Software.

**Supplementary Table 1.** Associations of vascular and metabolic indices with natriuretic peptides

|  | All Subjects (n=348) | | | Females (n=192) | | | Males (n=156) | | |
| --- | --- | --- | --- | --- | --- | --- | --- | --- | --- |
|  | ANP | BNP | CNP | ANP | BNP | CNP | ANP | BNP | CNP |
| BMI  Waist  Systolic BP  Diastolic BP  Pulse pressure  hsTroponin  Renin  Aldosterone  Plasma albumin  Plasma urate  Plasma creatinine  eGFR  ɣ glutamyltransferase  HOMA  HbA1c  Total Cholesterol  Chol/HDL ratio  Triglycerides  Haemoglobin  Haematocrit | -0.03  -**0.14**†  0.03  0.00  0.05  -**0.15**†  -**0.19**†  -**0.11***  -**0.15**†  -**0.23**†  -**0.25**†  -0.05  -**0.27**†  -**0.19**†  -0.13  -**0.11***  **-0.20**†  **-0.15**†  -**0.33**†  -**0.31**† | -0.07  -**0.18**†  -0.03  -0.05  0.00  -**0.11***  -**0.20**†  -**0.11***  -**0.23**†  -**0.25**†  -**0.28**†  -0.05  -**0.29**†  -**0.26**†  -0.06  -**0.16**†  -**0.26**†  -**0.22**†  -**0.34**†  -**0.31**† | 0.06  **0.16**†  **0.11***  **0.13***  0.04  **0.27**†  **0.19**†  0.02  **0.17**†  **0.25**†  **0.21**†  0.10  **0.28**†  **0.21**†  0.03  0.09  **0.21**†  **0.17**†  **0.32**†  **0.31**† | **-**0.07  **-**0.11  0.04  0.04  0.05  0.01  -**0.16***  -**0.16***  -0.12  -**0.20**†  -0.13  0.13  -**0.21**†  -**0.22**†  -0.18  -0.09  -0.13  0.00  **-0.21**†  **-0.18*** | -0.12  -**0.15***  -0.05  -0.08  -0.00  -0.01  -**0.19***  -**0.20**†  **-0.19**†  -0.14  -0.14  0.14  -**0.21**†  -**0.28**†  -0.13  -0.14  -**0.16***  -0.09  -**0.26**†  -**0.24**† | 0.10  0.10  0.13  0.14  0.08  **0.15***  **0.16***  0.07  0.12  0.13  -0.02  0.01  **0.22**†  **0.20**†  0.04  0.12  0.13  0.13  **0.28**†  **0.29**† | 0.03  -0.01  0.10  0.03  0.08  -0.07  -**0.26**†  -0.10  -0.08  -0.01  -0.05  0.04  -0.16  -**0.17***  -0.04  -0.10  -0.13  -**0.19***  -**0.23**†  -**0.23**† | -0.01  -0.04  0.10  0.06  0.04  0.01  -**0.21***  -0.03  -0.15  -0.06  -0.07  0.07  -**0.19***  -**0.26**†  0.03  -0.14  -**0.24**†  -**0.27**†  -0.14  -0.11 | 0.04  0.05  -0.01  0.07  -0.05  **0.16***  **0.19***  -0.06  0.08  0.09  0.08  -0.08  **0.16***  **0.20***  -0.01  0.02  0.11  0.04  0.10  0.08 |

* *P<0.05*

† *P<0.001*

**Supplementary Table 2.** Associations between echocardiography parameters and natriuretic peptides

|  | All Subjects | | | Females | | | Males | | |
| --- | --- | --- | --- | --- | --- | --- | --- | --- | --- |
|  | ANP | BNP | CNP | ANP | BNP | CNP | ANP | BNP | CNP |
| Stroke volume ‡ (303)  LA area ‡ (265)  LVEDV ‡ (303)  LVESV ‡ (303)  LV mass ‡ (276)  Ejection Fraction (294)  E/A (309)  E/e’ (301)  LVWEDWS ‡ (291)  LV elastance ‡ (303)  Arterial elastance ‡ (303) | -0.01  **0.18**†  -0.01  -0.01  -0.06  0.12  **0.14***  0.06  0.11  0.00  0.02 | 0.05  **0.15***  0.02  -0.05  -0.05  **0.17**†  **0.22**†  0.04  -0.01  0.05  -0.02 | -0.06  -0.07  -0.03  0.05  **0.13***  -**0.17**†  -**0.17**†  -0.01  -0.04  -0.01  0.09 | 0.06  **0.22**†  0.05  0.02  0.04  0.07  **0.24**†  0.05  0.11  0.01  0.00 | **0.17***  **0.20***  0.08  -0.06  0.12  0.15  **0.33**†  0.05  -0.12  0.06  -0.11 | -**0.22**†  -0.13  -**0.17***  -0.06  0.00  -0.08  -**0.25**†  -0.06  -0.04  0.08  **0.19*** | **0.17***  **0.21***  **0.22***  **0.19***  0.10  -0.03  0.01  0.03  0.07  -**0.17***  -0.12 | 0.14  0.14  **0.21***  **0.19***  0.01  0.02  0.08  -0.04  0.08  -0.12  -0.06 | -**0.21***  0.03  -**0.21***  0.10  0.01  -0.05  -0.09  0.11  -0.08  0.08  **0.17*** |

* *P<0.05*

† *P<0.001*

‡ Indexed to body surface area. Bracketed values indicate number of subjects.

**Supplementary References:**

1. Chantler PD, Lakatta EG. Arterial-ventricular coupling with aging and disease. Front Physiol 2012; 3:90
2. Abd-El-Aziz TA. Noninvasive prediction of left ventricular end-diastolic pressure in patients with coronary artery disease and preserved ejection fraction. Can J Cardiol 2012; 28:80-86
3. Iwanaga Y, Nishi I, Furuichi S, Noguchi T, Sase K, Kihara Y, Goto Y, Nonogi H. B-type natriuretic peptide strongly reflects diastolic wall stress in patients with chronic heart failure: Comparison between systolic and diastolic heart failure. J Am Coll Cardiol 2006; 47:742-748
